# Preface to special topic on games in control systems

**DOI:** 10.1093/nsr/nwaa118

**Published:** 2020-06-27

**Authors:** Ji-Feng Zhang

**Affiliations:** Key Laboratory of Systems and Control, Academy of Mathematics and Systems Science, Chinese Academy of Sciences, China; Guest Editor of Special Topic of NSR

In the past century, we have witnessed the glorious achievements of control science in our daily life, such as industry, transportation, aeronautics and astronautics, and so on. Traditionally, a control system is a set of mechanical or electronic devices, control of which usually deals with objects of passive nature. However, with the development of advanced sensing and communication technologies, ‘intelligent’ agents with not only execution and communication capabilities but also active objects are involved. In this case, traditional control methods may not be applied directly. To deal with active objects in control systems, game concepts and tools may be essential in the control framework. Due to the tremendous application foreground in various fields, including society, economics, artificial intelligence, etc., game-based control has inspired more and more interest among the control community.

To reflect the recent developments in the field of games in control systems, this special topic of the *National Science Review* presents four timely technical perspectives, a review and an interview.

The first perspective, by Zhang *et al*., introduces a new class of control systems referred to as game-based control systems that involve multiple active agents whose behaviours are driven by both physical laws and their own rational pursuits, and points out some new theoretical problems with potential applications, especially to man–machine integration systems. The second perspective, by Shamma, discusses the connections between game theory and control systems with representative examples, especially in the area of game-theoretic learning. The third perspective, by Cheng *et al*., shows the application and superiority of game theoretic control in optimization problems using a framework for optimization of networked systems via a game theoretic control-based approach. The fourth perspective, by Cao, discusses merging game theory and control theory in the area of artificial intelligence and autonomy, and expresses great expectations on this emerging research direction.

Modern control systems are featured by their hierarchical structure composed of cyber, physical and human layers. The review ‘Dynamic games for secure and resilient control system design’ by Huang *et al*. provides a multilayer perspective towards increasingly complex and integrated control systems, and offers an extensive overview of recent research directions on using game-theoretic methods to understand the fundamental trade-offs of robustness, security and resilience.

Control community has recently witnessed a rapidly growing interest in the application of game-theoretic concepts and tools in research on control, multi-agent systems, and networks. In the interview with NSR, Tamer Başar introduces the recently emerging role of game theory in control and networking research, how it broadens the territorial boundaries of control into disciplines outside engineering, and the opportunities and challenges that lie ahead.

As the guest editor, I would like to thank all of the authors, the reviewers, and the editorial staff for their contributions to this special topic. Games in control systems have attracted a great deal of attention in the control community, and there are still many fundamental and challenging problems that need to be investigated. I hope this special topic can provide useful information and promote research in this rapidly developing field.

